# Genetic diversity of 10 indigenous chicken ecotypes from Southern Highlands of Tanzania based on Major Histocompatibility Complex-linked microsatellite LEI0258 marker typing

**DOI:** 10.3382/ps/pez076

**Published:** 2019-03-16

**Authors:** Pius L Mwambene, Martina Kyallo, Eunice Machuka, Dedan Githae, Roger Pelle

**Affiliations:** 1 Tanzania Livestock Research Institute (TALIRI) - Uyole, Department of Research and Development, P.O. Box 6191, Mbeya, Tanzania; 2 Biosciences eastern and central Africa International Livestock Research Institute (BecA-ILRI) Hub, Capacity Building Unit, P.O. Box 30709-00100, Nairobi, Kenya

**Keywords:** characterization, genetic diversity, indigenous chicken ecotype, LEI0258 marker, Southern Highlands of Tanzania

## Abstract

Unraveling the genetic diversity of livestock species is central to understanding their value and importance for conservation and improvement in diverse production environments. In developing countries, information on genetic attributes of many livestock species is unfortunately scanty to support well-informed decision-making upon relevant management strategies. This study aimed at investigating allelic variability, genetic diversity, and genetic relationships of 10 indigenous chicken ecotypes from Southern Highlands of Tanzania using the Major Histocompatibility Complex-linked LEI0258 marker. A total of 400 DNA samples, 40 per ecotype, were genotyped by capillary electrophoresis. Thirty different alleles with sizes ranging from 197 to 569 bp were determined. The number of alleles ranged from 17 (Itunduma) to 21 (Mbeya), with an average of 19.20 alleles per ecotype. Allelic polymorphism was further evaluated through genotyping by Sanger sequencing. Thirty-three DNA samples with different fragment sizes were re-amplified and their alleles sequenced to depict polymorphism based on a combination of two repeat regions at 12 and 13 bp, respectively, and flanking regions with SNP and indels. The repeat region at 13 bp appeared 1 to 28 times, whereas the region at 12 bp appeared 3 to 19 times in all sequenced fragments. The numbers of indels and SNP determined were 7 and 9, respectively. From capillary electrophoresis, the Chunya and Msimbazi ecotypes exhibited the highest genetic diversity (0.937), whereas the lowest value (0.910) was observed from the Mbarali ecotype, with an average of 0.925. The Namtumbo and Wanging’ombe ecotypes showed high inbreeding coefficients (F_IS_ > 0.05), whereas a high excess heterozygote value (F_IS_ = –0.098) was observed from the Njombe ecotype. Two percent of the genetic diversity was due to differences among ecotypes, and the rest was due to differences among individuals within the ecotypes. Despite the overall low genetic differentiation, both fragment and sequencing analyses depicted a high allelic and genetic variability across 10 chicken ecotypes. These results therefore, underscore the importance of establishing appropriate conservation and management strategies to capitalize on observed variability and maintain genetic flexibility across diverse production environments.

## INTRODUCTION

Knowledge-based management of animal genetic resources **(AnGR)** is essential to answer the current agricultural, socioeconomic, and environmental challenges in the livestock development agenda. Consequently, characterization of AnGR constitutes one of the key priorities on appropriate management practices and is one of the key strategies for a global plan of action for the management of AnGR (FAO, 2007). Characterization is largely an important strategy in developing countries, where there is inadequate information regarding what and how to conserve, produce, and select among AnGR, including rural chicken ecotypes. Rural chicken ecotypes make a significant contribution largely to cultural matters, poverty alleviation, and household food and nutrition security in many developing countries, including Tanzania (Gueye, 2002; Alders and Pym, 2009).

In Tanzania, the history of introduction and dispersal of indigenous chickens countrywide is a subject of intense debate and speculation among scholars. However, sociocultural, linguistic, archaeological, and historic data suggest multiple sources of introductions over time and several dispersal routes towards and within the country. Molecular genetics information in Africa supports these observations and, in addition, suggests possible Asian centers of origin for African domestic chickens, including South Asia and Island Southeast Asia (Mwacharo et al., 2013).

In Tanzania, indigenous chickens (**IC**) form an integral part of highly variable agro-ecological zones and farming systems, and are the most adaptable and geographically widespread livestock. Currently, there are about 84.6 million chickens, producing approximately 4.15 billion of eggs and 99,540 metric tons of meat annually (Ministry of Livestock and Fisheries, 2016). These products are most preferred and considered to be tastier, safer, healthier, and having more quality than any other animal products. About 80% of these products are from IC stocks (Lwelamira et al., 2008; Ministry of Livestock and Fisheries, 2016).

Indigenous chickens are claimed to possess unique adaptive traits that help them survive and reproduce under harsh climatic, nutritional, and management conditions typically associated with low input–output production systems (Mwacharo et al., 2006, 2007; Ngeno et al., 2015). Despite the majority of IC populations being nondescript in phenotype and genotype in the country, some specific ecotypes are presently locally recognized based on indigenous knowledge and geographic location. A few ecotypes have further been sparsely evaluated on phenotypic, production, and genetic attributes based mainly on neutral microsatellite markers (Msoffe et al., 2005; Guni and Katule, 2013; Lyimo et al., 2013). The findings from these studies indicate significant variations among IC ecotypes on phenotypic, production, and genetic attributes within and among populations. This preliminary observation can, therefore, be capitalized to improved production through selective breeding. Variations on production and reproductive performance among locally recognized ecotypes have also been reported from other African countries (Tadelle et al., 2003; Ngeno et al., 2015). A few Tanzanian ecotypes have further been reported to be resistant or tolerant or both to endemic tropical diseases and parasites (Msoffe et al., 2006; Lwelamira et al., 2008). However, the inherent genetic diversity, structure, and the extent to which many locally recognized ecotypes are genetically distinct have yet to be determined. Lack of this information usually interferes with appropriate decision-making on relevant conservation and improvement strategies, and, therefore, threatening the continued existence of useful AnGR. Detailed knowledge on genetic diversity, distinctiveness, and relationships in combination with phenotypic and production attributes is essential for establishing development and conservation priorities and strategies (Caballero and Toro, 2002).

Globally, the LEI0258 microsatellite marker located within the Major Histocompatibility Complex (**MHC**) region has proven to be useful for the analysis of population genetic diversity, structure, distinctiveness, and relationships (Izadi et al., 2011; Chang et al., 2012). The marker has widely been used for genetic characterization of chicken populations worldwide, including 2 Tanzanian chicken ecotypes (Lwelamira et al., 2008). Unfortunately, no more studies have been conducted in Tanzania to establish the genetic diversity, structure, and relationships of IC ecotypes based on MHC-linked LEI0258 marker typing. This highly variable marker is located within the MHC region and is useful in showing the variability of this region. The LEI0258 marker has an atypical variable number of tandem repeats (**VNTR**), and its alleles show high polymorphism with large numbers, large size ranges, and composition (Fulton et al., 2006; Chang et al., 2012).

The LEI0258 marker is characterized by the repetition of 2 tandem and conserved short sequences of 12 and 13 base pairs (**R13/R12** regions) plus several sequence polymorphisms in the flanking regions (SNP and indels: small insertions and deletions), which allow easy identification of each allele using direct polymerase chain reaction **(PCR)** and sequencing (Han et al., 2013). It is the combination of the 2 repeats and the indels that determines the allele sizes. The MHC has important biological functions associated with immunity, design of effective vaccines, reproductive success, and production traits of domestic animals and is useful in elucidation of disease-associated studies (Bernatchez and Landry, 2003; Hoque et al., 2011; Izadi et al., 2011; Chang et al., 2012; Suzuki et al., 2012). This study, therefore, aimed at establishing allelic and genetic diversity, and relationships between 10 different IC ecotypes from the Southern Highlands of Tanzania based on MHC-linked LEI0258 marker typing. The results from this study in combination with information on their phenotypic and production attributes form a preliminary database based on which further investigation, future comparison, production improvement, and well-informed conservation strategies can be made.

## MATERIALS AND METHODS

### Blood Sample Collection and Total DNA Extraction

**Figure 1.**
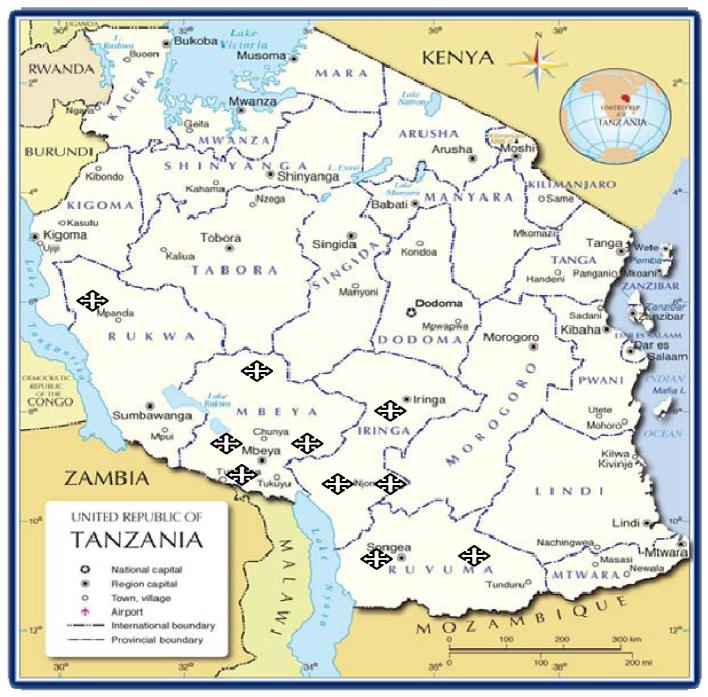
A map of Tanzania showing selected areas for blood samples collection Key: shows districts with different agro-ecological zones where blood samples were collected.

Blood sample was collected from a total of 400 IC from 10 districts in the Southern Highlands of Tanzania (40 samples per district). Each district represented an independent IC ecotype (Figure 1). A brief description of the phenotypic and production attributes and geographical location of selected ecotypes is indicated in Table 1. To reduce the probability of sampling genetically related birds, 5 free-ranging birds were randomly sampled per village, at most one mature chicken per household located at least 0.5 km away from the other. The blood sample was collected from chickens' wing veins and about 120 μL of blood per bird spotted onto the Whatman FTA Classic Card, as recommended by the supplier (GE Healthcare Life Sciences, North America 1-800-WHATMAN) for sample storage until DNA isolation. Both cockerels and hens were involved in blood sample collection in order to have an equal representation of both sexes per studied population.

**Table 1.** Some phenotypic attributes of selected indigenous chicken ecotypes from Southern Highlands of Tanzania.

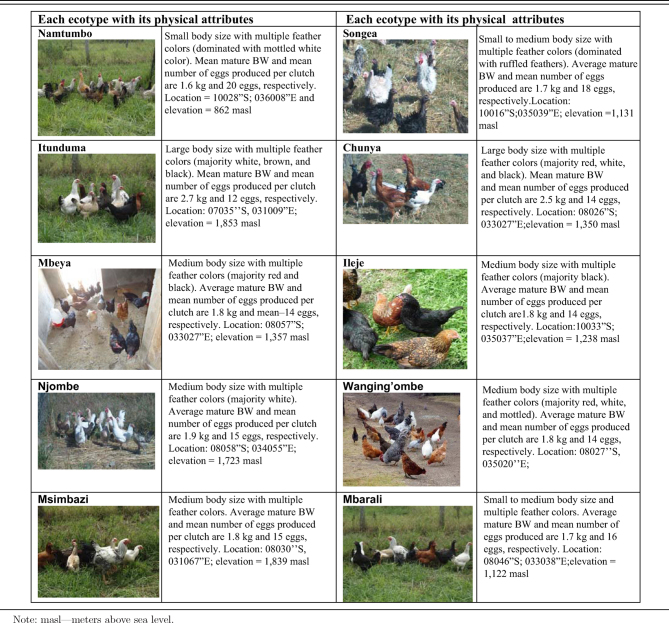

Total DNA was purified from FTA discs using an FTA Purification Reagent (GE Healthcare Life Sciences) as per the FTA manufacturer's instruction. One FTA disc (of 1.2 mm in diameter) was punched per sample and purified accordingly. The DNA was eluted from the FTA discs in 50 μL of milliQ H_2_O after boiling at 95°C for 5 min. The quantity and quality of the extracted DNA was checked using the NanoDrop 2000C spectrophotometer (Thermo Fisher Scientific, Waltham, MA) and 0.8% agarose gel electrophoresis. Eluted DNA from each sample was subsequently diluted to a working concentration of 20 ng/μL using milliQ H_2_O.

### PCR Amplification and Genotyping Using LEI0258 Microsatellite Marker

Polymerase chain reaction amplification was done using the primers LEI0258-F: **5′-CACGCAGCAGAACTTGGTAAGG-3′** and LEI0258-R: **5′-AGCTGTGCTCAGTCCTCAGTGC-3′** (McConnell et al., 1999; Fulton et al., 2006). The reaction recipe consisted of 40 ng of DNA, 0.1 μM of each primer, 1X Bioneer AccuPower PCR PreMix, and 3.4 μL of milliQ water added to a final volume of 10 μL. The PCR conditions used included 94°C for 3 min, followed by 30 cycles of 94°C for 45 s, 63°C for 1 min, 72°C for 2 min, and a final extension at 72°C for 20 min. The forward LEI0258 primer was tagged with the fluorescent dye PET at the 5′ end. PCR products were resolved in 2% agarose gel for verification of amplification. The gels were exposed to UV light to reveal the qualities and sizes of amplicons, by comparing with the O’GeneRuler 1 kb Plus DNA Ladder (Thermo Fisher Scientific).

For capillary electrophoresis, each PCR product was mixed with 8.9 μL of Hi-Di formamide and 0.1 μL of fluorescent-labeled GeneScan-500 LIZ size standard (Applied Biosystems, Warrington, UK). The mixture was denatured in a PCR machine at 95°C for 3 min and snap-chilled on ice for 5 min. The products were then electrophoresed using an ABIPRISM 3730xl automated sequencer (Applied Biosystems, Foster City, CA). Allele size scoring was performed twice, including the optimization stage, using the GeneMapper software version 4.1 (Applied Biosystems, Foster City, CA) to ascertain the correct size and number of alleles.

### Sequencing for Fine-analysis of Fragment Sizes and DNA Composition

Allelic polymorphism was further analyzed by Sanger sequencing. A total of 33 out of 400 DNA samples with different fragment sizes were amplified and sequenced successfully. One previously identified allele by capillary electrophoresis was represented by at least one DNA sample to depict the repeat regions with a combination of motifs or fragments (repeats 12 and 13) and flanking regions with SNP and indels. Homozygous samples were selected when possible, but heterozygous samples were used for DNA with alleles in heterozygote forms. T7 and SP6 promoter tagged-LEI0258 primers used included Forward: 5′-TAA TAC GAC TCA CTA TAG GGC ACG CAG AAC TTG GTA AGG-3′and Reverse: 5′-ATT TAG GTGACA CTA TAA GCT GTG CTC AGT CCT CAG TGC-3′ (with the underlined T7 and SP6 promoter sequences). Therefore, a subset of DNA samples in both homozygous and heterozygous forms and with private alleles were selected for amplification and sequencing. The PCR products were tagged with T7 and SP6 to enable direct sequencing of amplicons. The amplifications were performed in a 50 μL reaction volume containing 60 ng of DNA, 0.1 μM of both primers, 1X Bioneer AccuPower PCR PreMix, and 19 μL of milliQ water. The PCR cycling conditions were as for the genotyping profile with 5 additional cycles. The PCR products were purified with the QIAquick PCR Purification Kit (QIAGEN, Globalsave Limited, Uk) and QIAquick Gel Extraction Kit (QIAGEN, Globalsave Limited, Uk) for homozygous and heterozygous alleles, respectively. Sanger sequencing was carried out by the Bioneer Sequencing Service (Korea).

### Population (genotypic) Data Analysis

The number of alleles and expected and observed heterozygosity values were calculated using the GenAlEx 6.41 software (Peakall and Smouse, 2006). Overall genotypes and polymorphic information content **(PIC)** were assessed using the PowerMarker Software V.3.25 (Kejun et al., 2005). The GenAlEx 6.41 software was also used to test for conformity to Hardy–Weinberg equilibrium **(HWE)** and evaluate the heterozygote deficiency or excess per ecotype and pooled sample (Raymond and Rousset, 1995).

Genetic variations within and between populations were also established by analysis of molecular variance (**AMOVA**) using the GenAlEx 6.41 software (Peakall and Smouse, 2006). Standard genetic distances (D_A_) (Nei et al., 1983) between ecotypes were assessed based on allele frequencies using the GenAlEx 6.41 software. Principal component analysis (**PCA**) was also performed with the GenAlEx 6.41 software using allele frequencies in order to assess genetic relationships among the ecotypes. The first 2 principal components (**PC**) were used to identify possible population clusters.

### Sequence Data Analysis

Complete DNA sequences of LEI0258 alleles of selected samples and reference sequences retrieved from the GenBank database were aligned using the Clustal W function of the MEGA6 software and blasted against corresponding accessions in the NCBI GenBank database (Tamura et al., 2013). The aim of blasting was to ascertain sequenced alleles if they were novel to Tanzanian chicken populations or common elsewhere globally. A summary table was used to indicate polymorphisms at repeats (R13/R12) and flanking regions of all sequenced alleles based on conserved regions.

## RESULTS

### Allelic Variability from Capillary Electrophoresis

Analysis of allelic variability by capillary electrophoresis revealed a total of 30 different alleles across chicken ecotypes, with sizes ranging from 197 to 569 bp. Four private alleles were detected across the ecotypes. Each private allele was specific to only one ecotype: allele 569 (Itunduma), 465 (Songea), 460 (Namtumbo), and 440 bp (Msimbazi). The frequencies of private alleles were low, below 5% each. On the other hand, 9 alleles (with their frequencies in parentheses): 221 (10.63%), 315 (9.88%), 327 (8.13%), 209 (8.00%), 300 (7.38%), 363 (5.63%), 277 (5.50%), 426 (5.50%), and 263 (5.38%) were the most frequent across chicken ecotypes. A total of 8 alleles were common to all 10 ecotypes, and 16 alleles were shared by at least 2 chicken ecotypes. The number of alleles per ecotype ranged from 17 (Itunduma) to 21 (Mbeya), with an average of 19.20 alleles per ecotype (Table 2). However, the Chunya ecotype had the highest effective number of alleles (13.34), and the Mbarali ecotype had the lowest (9.91) (Table 5, shown later).

**Table 2.** Frequencies of LEI0258 alleles in 10 Tanzanian chicken ecotypes.

	Ecotypes											
Allele	CH	IL	IT	MBR	MB	MSB	NJ	NM	SG	WG	Overall freq	Pop
**197**	0.000	0.013	0.000	0.025	0.038	0.000	0.013	0.063	0.050	0.013	0.0213	7
**209**	0.113	0.063	0.050	0.025	0.100	0.038	0.188	0.063	0.125	0.038	0.0800	10
**221**	0.025	0.213	0.163	0.025	0.063	0.138	0.125	0.038	0.063	0.213	0.106	10
**239**	0.013	0.000	0.000	0.000	0.013	0.013	0.000	0.000	0.000	0.000	0.038	3
**245**	0.000	0.000	0.013	0.013	0.000	0.063	0.025	0.000	0.000	0.000	0.0113	4
**253**	0.050	0.000	0.025	0.025	0.013	0.038	0.050	0.100	0.000	0.013	0.0313	8
**263**	0.050	0.038	0.063	0.163	0.075	0.038	0.013	0.038	0.000	0.063	0.0538	9
**277**	0.038	0.075	0.050	0.000	0.075	0.100	0.038	0.000	0.075	0.100	0.0550	8
**289**	0.025	0.025	0.000	0.013	0.013	0.013	0.038	0.113	0.100	0.025	0.0363	9
**300**	0.050	0.038	0.075	0.025	0.163	0.075	0.113	0.038	0.138	0.025	0.0738	10
**312**	0.050	0.013	0.025	0.075	0.025	0.038	0.013	0.100	0.025	0.025	0.0388	10
**315**	0.050	0.063	0.150	0.113	0.100	0.050	0.075	0.163	0.100	0.125	0.0988	10
**325**	0.038	0.088	0.025	0.000	0.038	0.013	0.063	0.013	0.000	0.025	0.0300	8
**327**	0.088	0.075	0.075	0.138	0.025	0.113	0.063	0.075	0.063	0.100	0.0813	10
**340**	0.025	0.013	0.000	0.025	0.000	0.000	0.000	0.000	0.013	0.000	0.0075	4
**351**	0.063	0.113	0.038	0.013	0.038	0.025	0.038	0.038	0.038	0.038	0.0438	10
**363**	0.100	0.075	0.113	0.113	0.038	0.013	0.013	0.050	0.025	0.025	0.0563	10
**375**	0.000	0.000	0.000	0.025	0.000	0.025	0.038	0.013	0.013	0.000	0.0113	5
**385**	0.063	0.025	0.000	0.000	0.025	0.075	0.025	0.025	0.038	0.063	0.0338	8
**397**	0.013	0.013	0.063	0.025	0.025	0.050	0.013	0.000	0.000	0.000	0.0200	7
**411**	0.013	0.025	0.050	0.000	0.050	0.000	0.000	0.025	0.075	0.000	0.0238	6
**426**	0.138	0.038	0.013	0.138	0.063	0.075	0.038	0.000	0.013	0.038	0.0550	9
**440**	0.000	0.000	0.000	0.000	0.000	0.013	0.000	0.000	0.000	0.000	0.0013	1
**450**	0.000	0.000	0.000	0.000	0.013	0.000	0.025	0.000	0.013	0.025	0.0075	4
**460**	0.000	0.000	0.000	0.000	0.000	0.000	0.000	0.013	0.000	0.000	0.0013	1
**465**	0.000	0.000	0.000	0.000	0.000	0.000	0.000	0.000	0.013	0.000	0.0013	1
**472**	0.000	0.000	0.000	0.013	0.000	0.000	0.000	0.013	0.000	0.000	0.0025	2
**485**	0.000	0.000	0.000	0.000	0.013	0.000	0.000	0.013	0.000	0.013	0.0038	3
**497**	0.000	0.000	0.000	0.013	0.000	0.000	0.000	0.013	0.025	0.038	0.0088	4
**569**	0.000	0.000	0.013	0.000	0.000	0.000	0.000	0.000	0.000	0.000	0.0013	1
**Na**	19	18	17	19	21	20	20	20	19	19	19.20	

Notes: CH = Chunya; IL = Ileje; IT = Itunduma; MBR = Mbarali; MB = Mbeya; MSB = Msimbazi; NJ = Njombe; NM = Namtumbo; SG = Songea; WG = Wanging’ombe; freq = frequency; and Pop = number of populations sharing the allele.

### Allelic Variability by Sanger Sequencing

From Sanger sequence analysis, 2 levels of polymorphisms were considered, i.e., two repeat regions or motifs: R13 (CTATGTCTTCTTT) and R12 (CTTTCCTTCTTT) and indels and SNP along both flanking regions. The polymorphisms observed in the present study were 7 deletions (indels) and 9 SNP in the upstream and downstream regions. The deletions of TT were observed at positions –29 to 30 bp, i.e., 29 bp before the R13 repeats along the upstream region. On the other hand, no large deletion or insertion (indel) was observed along the downstream sequence. The conserved region, which could make an indel along the downstream sequence, consisted of 8 bp (ATTTTGAG) and was located at positions +23 to +30. The sizes of sequenced alleles, which reflected the combination of the repeats/motifs and indels for sequenced alleles, ranged from 249 to 552 bp. Numbers of repeat regions (R13 and R12) differed across sequenced alleles. R13 appeared 1 to 28 times, whereas R12 appeared 3 to 19 times depending on the allele sizes. The upstream region was at positions −78 to −1, and the downstream region at 1 to 88 bp. The majority of sequenced alleles showed combinations of only one R13 motif with several R12 repeats (28 out of 33 combinations). The following R13 and R12 combinations were the most frequent across all 33 sequenced alleles: one with 13, one with 16, and one with 11 (Table 3).

**Table 3.** Polymorphisms identified by LEI0258 alleles in 10 selected indigenous chicken ecotypes.

			Position					Position							
			–30 to 29	−28	–11	Repeat motifs		5	(23 to 30)	33	39	46	GenBank accession references		
Chicken samples	Fragment size (bp, by genotyping)	Consensus size (bp, by sequencing)	TT	G	G	R13	R12	C	ATTTTGAG/Δ	Δ	A	T/A	% Identity	Reference number	Corresponding isolate, clone or haplotype
Chunya 26	312	305	–	–	–	9	3	–	**–**	A	–	A	100	KF535093.1	NYCAU-10
Songea 21b	300	295	**Δ**	A	–	1	11	–	–	A	–	A	99	MG495235.1	ShubiGemo_1H
Songea 12	300	295	**Δ**	A	**–**	1	11	**–**	**–**	A	**–**	A	99	MG518271.1	Cos116a
Chunya 1a	426	419	–	–	–	14	6	–	**–**	A	–	A	100	KF534951.1	NYCAU-50
Mbarali 18	327	321	–	–	–	1	13	–	**–**	A	–	A	99	MG495241.1	Dikuli_7H
Namtumbo 37	351	345	–	–	–	1	15	–	–	A	–	A	99	MG495244.1	Hugub_H9
Songea 21a	327	321	–	–	–	1	13	**–**	**–**	A	**–**	A	99	MG495241.1	Dikuli_7H
Wanging’ombe 2	300	295	**Δ**	A	–	1	11	–	**–**	A	–	A	100	MG495235.1	ShubGemo_1H
Wanging’ombe 15a	263	261	–	–	–	1	8	–	**–**	A	–	A	100	MG518288.1	Cen3a
Wanging’ombe 30	277	261	–	–	–	1	8	–	**–**	A	–	A	100	MG518288.1	Cen3a
Wanging’ombe 37	363	357	–	–	–	1	16	–	**–**	A	–	A	99	MG495245.1	Adane_9C
Wanging’ombe 39	289	273	–	–	–	1	9	–	**–**	A	–	A	100	KF534932.1	NYCAU-31
Itunduma 18	325	309	–	–	–	1	12	–	**–**	A	–	A	100	MG518283.1	Wes97b
Chunya 13	385	379	**Δ**	A	–	1	18	–	**–**	A	–	A	100	MG495248.1	Kumato_5H
Ileje 13	363	357	–	–	–	1	16	A	**–**	A	–	A	99	MG495245.1	Adane_9C
Mbarali 6	426	419	–	–	–	15	6	–	**–**	A	–	A	100	KF534951.1	NYCAU-50
Chunya 1b	426	419	–	–	–	15	6	–	–	A	–	A	100	KF534951.1	NYCAU-50
Mbeya 7	363	357	–	–	–	1	16	A	**–**	A	–	A	99	MG495245.1	Adane_9C
Itunduma 9	315	309	–	–	–	1	12	–	–	A	–	A	100	MG518283.1	Wes97b
Mbarali 10	363	357	–	–	–	1	16		**–**	A	–	A	99	MG495245.1	Adane_9C
Wanging’ombe 20	327	321	–	–	–	1	13	–	–	A	–	A	100	MG495241.1	Dikuli_7H
Mbeya 38	277	273	–	–	–	1	9	–	–	A	–	A	100	KF534932.1	NYCAU-31
Wanging’ombe 15b	263	261	–	–	–	1	8	–	–	A	–	A	100	MG518288.1	Cen3a
Itunduma 22–A1	569^1^	552	–	–	–	28	3	–	**–**	A	–	A	99	MG518262.1	Cen173a
Itunduma 22–A2	397	391	**Δ**	A	–	1	19	–	**–**	A		A	98	DQ239493.1	haplotype B1
Itunduma 22–B1	397	391	**Δ**	A	–	1	19	–	**–**	A	–	A	98	DQ239493.1	haplotype B1
Namtumbo 32–B2	385	381	–	–	–	1	18	–	**–**	A	–	A	100	MG518264.1	Cos8b
Msimbazi 18–B1	253	249	–	–	–	1	7	–	–	A	–	A	100	MG518284.1	Wes35b
Itunduma 6–B1	325	319	–	–	–	1	13	–	–	A		A	100	KF534944.1	NYCAU-43
Itunduma 40–B1	300	295	**Δ**	A		1	11	–	–	A	–	A	100	MG495235.1	ShubiGemo_1H
Wanging’ombe 1A1	363	357	–	–	–	1	16	–	**–**	A	–	A	99	MG495245.1	Adane_9C
Wanging’ombe 39–B2	327	321	–	–	–	1	13	–	**–**	A	–	A	99	MG495241.1	Dikuli_7H
Namtumbo 39–B1	289	273	–	–	–	1	9	–	**–**	A	–	A	100	KF534932.1	NYCAU-31

Note: ^1^Private allele: the allele appeared only in one population.

Δ: defines deletion compared with the reference sequence.

–: consistent with the reference sequence.

The sizes of alleles detected by capillary electrophoresis (fragment lengths) and sequencing (consensus sizes) did not exactly match. The size differences ranged from 2 to 17 bp. On the other hand, results from NCBI blasting of all sequenced alleles indicated 19 alleles to be 100% similar to their corresponding accessions from the GenBank database. However, similarities of 14 sequences against their corresponding accessions in GenBank were slightly less than 100% (Table 3). All the sequences were submitted to NCBI and later on provided with new accession numbers from the NCBI database. The accession numbers ranged from MG518290 to MG518322 (Table 4).

**Table 4.** Chicken DNA samples with sequences provided with accession numbers from NCBI.

S/No	Chicken sample identity	New accession numbers
1	Chunya_26	MG518290
2	Songea_21	MG518291
3	Songea_12	MG518292
4	Chunya_1a	MG518293
5	Mbarali_18	MG518294
6	Namtumbo_37	MG518295
7	Songea_21a	MG518296
8	Wanging’ombe_2	MG518297
9	Wanging’ombe_15a	MG518298
10	Wanging’ombe_30	MG518299
11	Wanging’ombe_37	MG518300
12	Wanging’ombe_39	MG518301
13	Itunduma_18	MG518302
14	Chunya_13	MG518303
15	Ileje_13	MG518304
16	Mbarali_6	MG518305
17	Chunya_1b	MG518306
18	Mbeya_7	MG518307
19	Itunduma_9	MG518308
20	Mbarali_10	MG518309
21	Wanging’ombe_20	MG518310
22	Mbeya_38	MG518311
23	Wanging’ombe_15	MG518312
24	Itunduma_22_A1	MG518313
25	Itunduma_22_A2	MG518314
26	Itunduma_22_B1	MG518315
27	Namtumbo_32_B2	MG518316
28	Msimbazi_18_B1	MG518317
29	Itunduma_6_B1	MG518318
30	Itunduma_40_B1	MG518319
31	Wanging’ombe_1_A1	MG518320
32	Wanging’ombe_39_B2	MG518321
33	Namtumbo_39_B1	MG518322

### Population Genetic Diversity Results

Analyses of data from capillary electrophoresis indicated overall PIC at this marker to be 0.935. The overall means of observed **(Ho)** and expected **(He)** heterozygosity were 0.898 ± 0.02 and 0.925 ± 0.00, respectively. The Njombe ecotype showed the highest Ho (1.00) and the Wanging’ombe ecotype showed the lowest Ho (0.775). The Chunya and Msimbazi ecotypes showed the highest He (0.937), whereas the Mbarali ecotype showed the lowest value (0.91). The Chunya, Ileje, Mbarali, Mbeya, Namtumbo, Songea, and Wanging’ombe ecotypes had lower Ho than He, whereas the reverse was true for the Njombe, Itunduma, and Msimbazi ecotypes (Table 5).

**Table 5.** Number of alleles, observed and expected heterozygosity, effective number of alleles, and Hardy–Weinberg equilibrium (HWE) status.

Sub-pop (N = 40)	TNA	Allele range	A_e_	H_o_	He	F_IS_ values	HWE deviation
Chunya	19	209 to 426	13.34	0.900	0.937	0.027	b^ns^
Ileje	18	197 to 426	10.32	0.900	0.915	0.003	b^ns^
Itunduma	17	209 to 569	10.88	0.950	0.920	–0.046	b***
Mbarali	19	197 to 497	9.91	0.900	0.910	–0.001	b^ns^
Mbeya	21	197 to 485	13.01	0.900	0.935	0.025	b***
Msimbazi	20	209 to 440	13.33	0.950	0.937	–0.027	b^ns^
Njombe	20	197 to 450	11.23	1.000	0.922	–0.098	b**
Namtumbo	20	197 to 497	12.08	0.825	0.929	0.101	b**
Songea	19	197 to 497	12.26	0.875	0.930	0.047	b*
Wanging’ombe	19	197 to 497	10.16	0.775	0.913	0.140	b***
**Mean**	**19.20 ± 3.6**		**11.66 ± 0.4**	**0.898 ± 0.02**	**0.925 ± 0.00**	**0.017 ± 0.022**	
**Entire population**	**30**	**197** to **569**		**0.8975**	**0.9388**	**0.030**	

Notes: N = number of samples per ecotype; TNA = total number of alleles; H_o_ = observed heterozygosity; H_e_ = expected heterozygosity; A_e_ = effective number of alleles; I = information index; F_IS_ = within-population inbreeding coefficient; b*, **, *** = populations not in HWE (*P* < 0.05; *P* < 0.01; *P* < 0.001); b^ns^ = populations with non-significant deviation from HWE.

The overall and average inbreeding coefficients (F_IS_ = 0.03 and 0.017, respectively) were low and, therefore, insignificantly different from zero. However, the Wanging’ombe and Namtumbo ecotypes showed high and positive F_IS_ values (0.140 and 0.101, respectively), which were significantly different from zero. On the other hand, the Njombe ecotype showed a high and negative F_IS_ value (–0.098), which was also significantly different from zero. The remaining ecotypes showed low F_IS_ values (<0.05), which were not significantly different from zero. Five ecotypes with a significant deviation from HWE exhibited deficient heterozygotes, except the Itunduma ecotype, which showed an excess heterozygote (Table 5).

### Distribution of Total Genetic Variation Among Chicken Ecotypes

**Figure 2.**
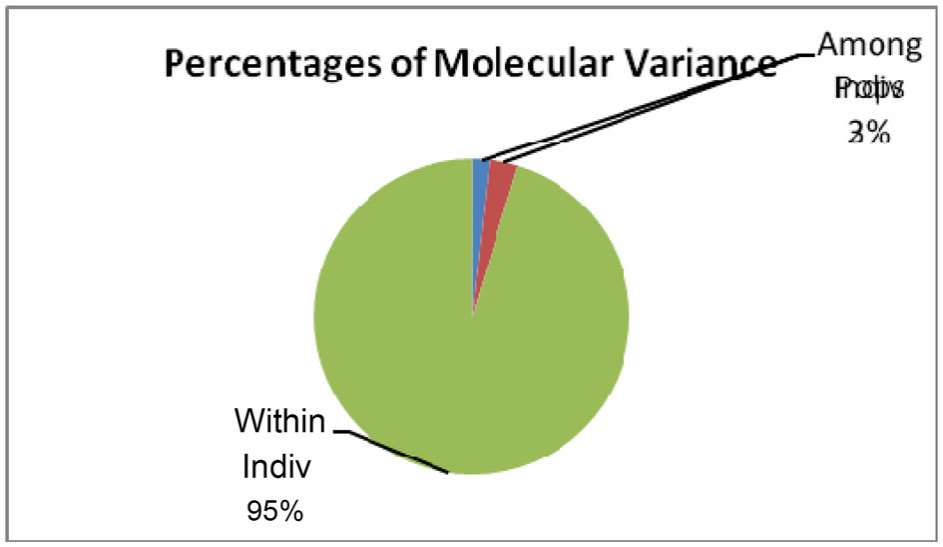
Analysis of molecular variance (AMOVA).

Results from AMOVA indicated 98% of the total genetic variation of studied ecotypes to be due to variations of individual birds within and across populations. Only 2% of the total genetic variation was due to variations among studied ecotypes (Figure 2).

### Genetic Distance and Relationships

**Figure 3.**
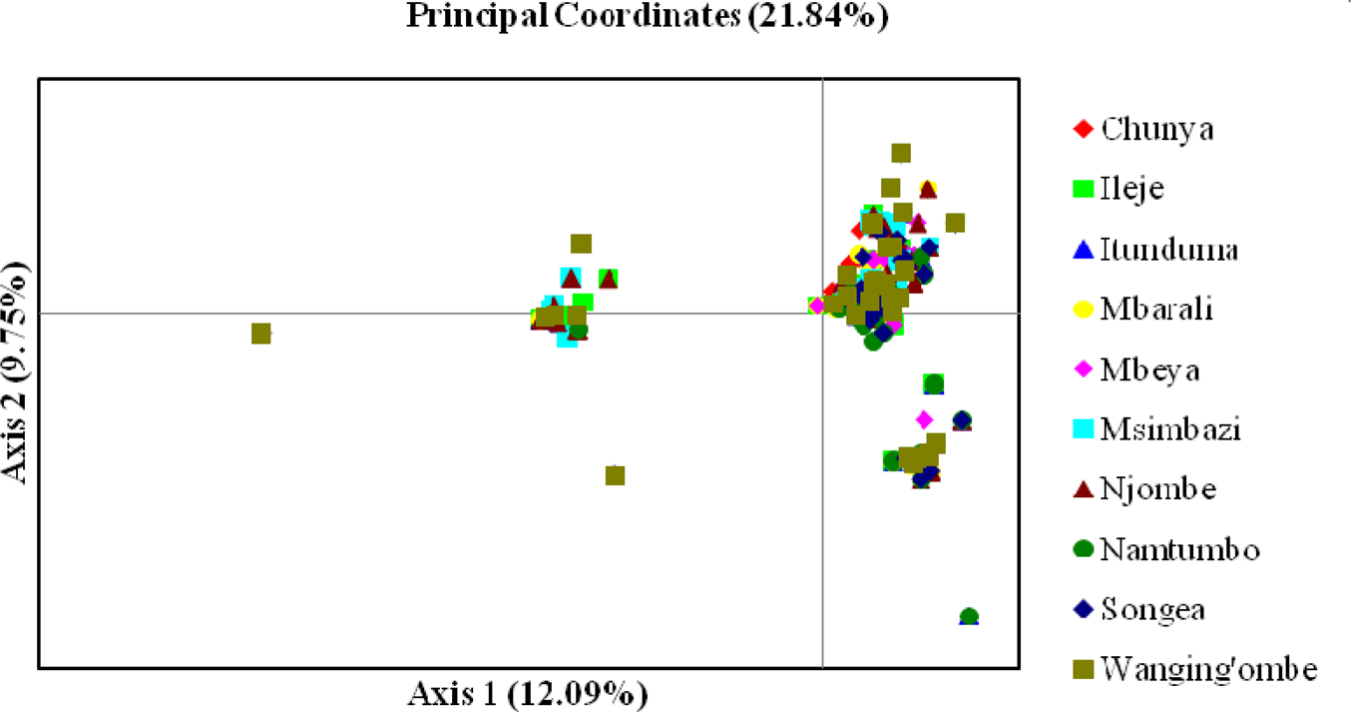
Principal components graph of the first 2 principal components from 10 chicken populations.

Table 6 summarizes pairwise standard genetic distances (D_A_) and gene differentiation (F_ST_) indices among studied ecotypes. Both D_A_ and F_ST_ indices ranged from 0.000 for the Mbeya and Songea ecotypes to 0.726 and 0.045 for the Mbarali and Njombe ecotypes, respectively. On the other hand, Figure 3 shows genetic relationships among ecotypes using PCA. The first two PC accounted for 21.84% of the total genetic variation. The first PC explained 12.09% and the second PC explained an additional 9.75% of the variation. Both components did not separate the 10 chicken ecotypes into distinct clusters. However, 3 roughly admixed sub-clusters were formed with a few individual outliers.

**Table 6.** Pairwise Nei unbiased genetic distances (D_A_ below diagonal) and fixation indices (F_ST_ above diagonal) between chicken ecotypes.

Ecotypes	1	2	3	4	5	6	7	8	9	10
1. Chunya	–	0.020^2^	0.020^1^	0.009ns	0.008ns	0.011^2^	0.013^1^	0.018^2^	0.017^2^	0.025^2^
2. Ileje	0.285	–	0.004ns	0.041^2^	0.018^2^	0.009^1^	0.014^1^	0.033^2^	0.023^2^	0.001ns
3. Itunduma	0.301	0.045	–	0.023^2^	0.008ns	0.010ns	0.016^1^	0.018^2^	0.017^2^	0.004ns
4. Mbarali	0.104	0.591	0.288	–	0.026^2^	0.026^2^	**0.045** ^2^	0.023^2^	0.043^2^	0.033^2^
5. Mbeya	0.141	0.250	0.108	0.381	–	0.011^1^	0.005ns	0.019^2^	**0.000ns**	0.019^2^
6. Msimbazi	0.184	0.111	0.132	0.369	0.172	–	0.012^1^	0.027^2^	0.017^1^	0.001ns
7. Njombe	0.187	0.162	0.206	**0.726**	0.054	0.152	–	0.022^2^	0.005ns	0.018^2^
8. Namtumbo	0.314	0.536	0.266	0.323	0.332	0.491	0.335	–	0.011^1^	0.025^2^
9. Songea	0.289	0.326	0.241	0.703	**0.000**	0.273	0.057	0.178	–	0.021^2^
10. W/ng’ombe	0.392	0.003	0.048	0.445	0.270	0.015	0.227	0.379	0.305	–

Note: ^1^F_ST_ values significantly different from zero (*P* ≤ 0.05).

^2^F_ST_ values significantly different from zero (*P* ≤ 0.01).

W/ng’ombe—Wanging’ombe.

## DISCUSSION

### Allelic Variability at LEI0258 Microsatellite Marker

This study aimed at assessing the allelic and genetic diversity as well as relationships of 10 chicken ecotypes from the Southern Highlands of Tanzania using the MHC-linked LEI0258 marker in order to ascertain the worthiness of these ecotypes for improvement and conservation. Genotyping by both fragment and Sanger sequencing analyses was employed.

From capillary electrophoresis, a total of 30 distinct alleles were scored from 10 selected ecotypes, with sizes ranging from 197 to 569 bp. Out of 30 alleles, 4 alleles were private and existed in only one ecotype each, whereas 8 alleles were observed in all 10 chicken ecotypes. Nine alleles were the most frequent across chicken ecotypes. The presence of many common and frequent alleles across chicken ecotypes suggests close relationships in all studied ecotypes. The minimum and maximum numbers of alleles per ecotype were 17 and 21, respectively. The large numbers and big range of allele sizes at this marker implicate high allelic polymorphism across studied ecotypes. Differences in allele numbers among ecotypes might be an indication of differences of chicken populations depending on their origins, dispersion, production environments, and level of interactions within and between ecotypes.

The average number of alleles per ecotype in the present study is slightly lower than the mean numbers reported previously for the Tanzanian Kuchi (22) and Medium (23) ecotypes (Lwelamira et al., 2008). Differences in the number of birds sampled per ecotype (40 vs. 88 birds) might be the reason for this observed discrepancy, since the number of alleles and heterozygosity values are sometimes influenced by sample sizes studied (Nei, 1978). Exposures to different production environments, level of interactions among ecotypes, and different origins might also be sources of the slight discrepancy observed. The numbers of alleles observed in the present study are within the range (20 to 27) of alleles reported in Kenyan native ecotypes (Ngeno et al., 2015), but they are higher than the 15 alleles that were reported in Brazilian chickens (Lima-Rosa et al., 2005), 13 alleles that were found in Korean native chickens (Piertney and Oliver, 2006), and 16 alleles that were reported in 6 Taiwan chicken breeds (Chang et al., 2012). On the other hand, the number is lower than 26 alleles observed in North American and European layer-type chickens (Fulton et al., 2006), 25 alleles reported in Iranian chicken populations (Nikbakht et al., 2013), and 25 alleles observed in two chicken breeds in Vietnam (Schou et al., 2007) at the same locus.

A total of 4 private alleles were detected in 4 ecotypes, one allele each. This observation is an indication of within-country ecotypes specificity, and could be used to distinguish them locally. The existence of different private alleles is an indication of the presence of MHC genetic diversity among ecotypes due to different origins and adaptation to different production environments. However, the frequencies of all 4 private alleles were generally low, thus implying the ecotypes to be genetically indistinct and sharing the majority of alleles observed. Low frequencies of private alleles normally do not warrant their application in population specificity evaluation, whereas sharing of many alleles among ecotypes is an indication of populations being either genetically related or being subjected to a similar directional selection, or the presence of a high rate of gene flows among populations.

From sequences analysis, 33 fragments indicated the R13 repeat to appear from one to 28 times and from 3 to 19 times for the R12 repeat. In addition, 16 polymorphisms were observed from the flanking (upstream and downstream) regions. The upstream region showed 7 insertions (indels) and 7 SNP, whereas the downstream region exhibited only 2 SNP. The TT indel position was at position –29 to 30 bp, i.e., 29 bp before the R13 repeats, instead of the –31 to 32 bp position, which was previously reported in the North American and European layer-type chickens (Fulton et al., 2006). On the other hand, no indel was observed along the downstream sequence. However, the conserved region was located at positions +23 to +30 and consisted of 8 bp (ATTTTGAG) instead of 7 bp, which was previously reported in the North American and European layer-type chickens (Fulton et al., 2006). Positions and sizes of all conserved regions for studied chicken ecotypes conformed well to those of Chinese IC populations (Piertney and Oliver, 2006; Han et al., 2013; Wang et al., 2014) but did not conform to the positions, regions, and sizes reported in North American and European layer-type chickens (Fulton et al., 2006) or in 80 different populations and lines found in Africa, Asia, and Europe (Chazara et al., 2013). Based only on LEI0258 allele compositions and not the entire MHC region, there is an indication of genetic similarities between Chinese and Tanzanian IC populations unlike the layer-type chicken populations found in North America and Europe. Genetic similarities between Chinese and Tanzanian IC at this marker might be associated with sharing of recent common ancestors, centres of origin, and high gene flows between the Asian and East African chicken populations. The number of repeats (motifs) and their respective combinations and the number of SNP and indels from the present study differ from the findings from the previous studies (Chazara et al., 2013; Han et al., 2013). This observation is an indication of genetic polymorphisms and evolution dynamics within and among chicken populations. However, the similarities or differences between Tanzanian and Chinese or European chicken breeds might also vary when the entire haplotypes or MHC region is considered since LEI0258 typing alone is not sufficient to depict comprehensively the variability of the MHC-*B* region. Furthermore, the mutation rate of LEI0258 is always a source of error in estimating MHC variation when using this marker alone (Fulton et al., 2016).

In this study, 14 sequenced alleles had corresponding accessions from GenBank (NCBI) with similarity levels slightly less than 100%. All these alleles might be novel for Tanzanian chicken populations. However, this observation needs further verification. On the other hand, the fragment lengths by capillary electrophoresis did not exactly match those obtained by sequencing for unclear reasons. Some environmental factors might have affected electrophoresis, or capillary electrophoresis might have shown false-positive results. Moreover, LEI0258 is a composite VNTR, which is characterized by repetition of 2 tandem and conserved short sequences of 13 and 12 bp (R13/R12 regions) plus several sequence polymorphisms in the flanking regions (SNP and indels). It is common for a compound microsatellite to contain different repeating units with the same size of the fragments. Therefore, fragment analysis alone without combining with sequence analysis at this marker may lead to poor or inadequate estimation of actual divergence among studied chicken ecotypes (Chang et al., 2012). A combination of both capillary electrophoresis and sequencing analyses provides more accurate evidence on actual genetic variability among chicken populations (Han et al., 2013).

### Genetic Variability at LEI0258 Marker

The genetic diversity observed across chicken ecotypes ranged from 91 (Mbarali) to 93.7% (Msimbazi and Chunya). A high level of genetic diversity observed in this study could be attributed to high antigenic diversity and other stresses that are prevailing in the free range production environments in which these chickens have evolved and are kept. The frequency of heterozygosity at the MHC is also expected to be higher in outbred populations with high gene flows and exposure to all kinds of infectious agents. Selected populations were managed extensively with free movements and high admixtures from different agro-ecological conditions.

The range of genetic diversity observed in the present study is relatively higher than that of 86.4 to 88.2% and 84 to 88%, which were reported in both the Kuchi and Medium ecotypes from eastern Tanzania (Lwelamira et al., 2008) and Kenyan local chicken populations (Ngeno et al., 2015), respectively. The present He values are almost similar to the value (91%) reported from one Vietnamese local chicken population (Schou et al., 2007), but significantly higher than those of 50 and 75% reported from two Brazilian local chicken populations (Lima-Rosa et al., 2005), at the same microsatellite marker.

The high gene diversity observed in the current and previous studies is consistent with the great phenotypic and production variability, which was previously reported in similar and other Tanzanian populations (Lwelamira et al., 2008; Guni and Katule, 2013). The combination of present results and previously documented phenotypic information provides robust evidence that can support well-informed decision-making on prioritization, development, and conservation. The high genetic diversity of native populations, which are usually subjected to varied production environments and challenges, also enhances the adaptability of the ecotypes to changing environments, market demand, and breeding goals (Notter, 1999). Moreover, the high within-populations genetic variation (98%) observed in the present study is an indication of ecotypes being composed of more heterogeneous birds. This observation is also evident from variable phenotypic attributes, which were documented before on the same ecotypes (Guni and Katule, 2013).

Nevertheless, the majority of the ecotypes had Ho values lower than He values, thereby pointing to a possible departure from random mating. This is evident from the fact that of the 10 populations studied 6 showed heterozygote deficiencies. This observation was further augmented by the presence of 6 out of 10 IC ecotypes with significant deviations from HWE at this locus. This observation might have resulted from limited random mating or mating between relatives, similar to what has been reported by many scholars on other livestock species (Rehman and Khan, 2009). Although it is difficult to envisage the exact basis of observed heterozygote deficiencies and deviations from HWE at this locus, they suggest it to be the presence of inbreeding, non-random mating, bias in sampling procedure, or population subdivision. They further imply the presence of selective disadvantage for certain heterozyogote combinations, especially in light of the strong association between MHC and disease resistance. On the other hand, the excess heterozygosity in some populations might be due to a recent introduction of novel cockerels from other breeds for crossbreeding with native birds at a preliminary level.

### Distinctiveness and Relationships of the Chicken Ecotypes

One of the main objectives of this study was to evaluate the genetic relationships of 10 promising IC ecotypes in order to determine if they merit to be considered as distinct ecotypes for conservation, promotion, and improvement. The evaluation was based on coefficients of genetic differentiation (F_ST_), standard genetic distance (D_A_), and PCA. The mean coefficients of genetic differentiation observed in this study demonstrated that only 2% of the total genetic variation was accounted for by between-ecotype differences, whereas the rest (98%) was attributed to differences among individuals within chicken ecotypes. These findings signify the overall genetic diversity of 10 selected ecotypes from the Southern Highlands chicken populations to be highly influenced by heterogeneities of individual birds within and across populations unlike differences between chicken ecotypes. The observed overall level of genetic differentiation among ecotypes was lower than that reported (4.8%) by Lyimo et al. (2013) in other Tanzanian local chicken ecotypes. However, the previous study employed 29 neutral microsatellite loci instead of the LEI0258 marker.

The majority of pairwise F_ST_ and D_A_ values were also relatively small, indicating low levels of genetic differentiation and distance, respectively, between the IC populations. Low genetic differentiation and distances between IC ecotypes are attributed to high gene flows, admixture, and interbreeding in the study area as well as sharing a common ancestry among the ecotypes. According to Laval et al. (2000), migration and admixture may exert a greater effect than mutation or drift on the reduction of genetic differentiation between populations. However, the Mbarali and Njombe ecotypes were relatively distant from each other unlike the other pairs within the Southern Highlands clusters, thus warranting further investigation. The distance barrier and isolation of the Mbarali ecotype due to its remoteness from other sites might be the reason for being relatively distant from the Njombe and other ecotypes.

The results from PCA supported the close relationships among the chicken ecotypes. These observations further signify that the IC ecotypes are highly admixed or share a common ancestry and that their separation might be only recent. The findings of the present study are consistent with the results that have been reported by Moioli et al. (2004) in the Italian cattle breeds. This study observed that individual animals from breeds that had no gene flow between them were clearly coherent in their respective clusters. But, breeds that had been admixed had some of their members mis-assigned away from their respective clusters.

## CONCLUSIONS

All 10 IC ecotypes depicted large genetic variations and hosted multiple and highly variable MHC-linked alleles. High allelic and genetic diversities observed at the MHC region of the studied chicken ecotypes support their claimed value of being hardy, adaptive, and resilient to various production environmental challenges, including diseases and parasites. This observation offers a basic step towards well-informed decision-making on relevant management strategies for improvement, development, and conservation without compromising the existence of each unique chicken genetic resource in its present environment. Routine assessment of genetic attributes of native AnGR is worthwhile for present and future uses. However, the utilization of the VNTR LEI0258 marker alone is only an indicator of MHC diversity because the mutation rate of the LEI0258 alleles might be a source of error in estimating variations with this marker.

## Statement of interest

The authors strongly declare that there are no conflicts of interest.
